# Anatomical localization and acupuncture pathway of the sphenopalatine ganglion in Sprague–Dawley rats: a microanatomical study

**DOI:** 10.3389/fneur.2025.1704434

**Published:** 2025-11-26

**Authors:** Yuechun Zhao, Lei Li, Mingjiang Yao, Xiaojing An, Jing Wang, Zhengyang Li, Hanxi Dai, Jinxin Dai, Lu Zhang

**Affiliations:** 1 Xiyuan Hospital of China Academy of Chinese Medical Sciences, Beijing, China; 2 Beijing Key Laboratory of Pharmacology of Chinese Materia, Institute of Basic Medical Sciences of Xiyuan Hospital, China Academy of Chinese Medical Sciences, Beijing, China

**Keywords:** sphenopalatine ganglion, pterygopalatine fossa, microscopic dissection, acupuncture pathway, head and facial diseases

## Abstract

**Background:**

The sphenopalatine ganglion (SPG) plays a pivotal role in regulating various head and facial diseases, such as cluster headaches, allergic rhinitis, and cerebral ischemia, through its neuroregulatory functions. Current interventions targeting SPG, have certain limitations, such as invasiveness and high cost. Acupuncture, which shows unique advantages, lacks standardized animal models for experimental research.

**Objective:**

To investigate the spatial anatomical localization of the SPG in Sprague–Dawley (SD) rats and to establish an effective acupuncture pathway for SPG.

**Methods:**

A total of 12 SPF-grade SD rats (6 males and 6 females) were randomly divided into dissection and acupuncture groups. The SPG was microanatomically localized relative to bony landmarks (e.g., sphenopalatine foramen, foramen rotundum). An acupuncture pathway was designed based on anatomical data and validated using digital X-ray fluoroscopy. Serial staining (HE staining, Nissl staining, immunofluorescence staining) was employed to analyze the neural architecture and status within the pterygopalatine fossa region.

**Results:**

The SPG was located laterally to the sphenopalatine foramen, with coordinates for males being *X* = +0.05 ± 0.03 mm, *Y* = −0.62 ± 0.08 mm, and for females *X* = +0.04 ± 0.01 mm, *Y* = −0.54 ± 0.06 mm. The acupuncture needle was inserted at the outer canthus, 0.8–1.0 cm from the eye’s lateral canthus, at a sagittal angle of 30–45° and a coronal angle of 45°, reaching a depth of approximately 1 cm. X-ray fluoroscopy confirmed that the needle tip successfully reached the SPG area. Serial staining results indicated a normal distribution of the neural network in this area and showed no significant structural damage caused by the acupuncture intervention.

**Conclusion:**

This study successfully established a precise anatomical coordinate system and a reproducible acupuncture pathway for the SPG in SD rats. These findings provide a technical foundation for experimental acupuncture interventions targeting SPG, which may be beneficial for treating head and facial diseases, such as cluster headaches and allergic rhinitis.

## 1 Introduction

The pterygopalatine fossa (PPF) is a small, cone-shaped anatomical space located at the center of the cranial base (1). As a pivotal neurovascular passage, it houses the sphenopalatine ganglion (SPG), the largest peripheral parasympathetic ganglion in the human body. The SPG integrates parasympathetic fibers from the facial nerve, sympathetic fibers from the superior cervical ganglion, and sensory fibers from the maxillary branch of the trigeminal nerve. It subsequently regulates lacrimal secretion, nasal mucosal vasomotion, and palatal sensation, playing a central role in ocular moisture regulation, nasal ventilation, and craniofacial pain conduction.

In recent years, with the advancement of neuroscience and minimally invasive techniques, the clinical significance of the PPF and SPG has been progressively elucidated. Evidence-based medicine indicates that SPG stimulation, as a neuroregulatory technique, has shown significant efficacy in various head and facial diseases, including cluster headaches (2), allergic rhinitis (3), and cerebral ischemia (4). SPG stimulation is primarily classified into invasive and non-invasive methods. Invasive interventions involve the surgical implantation of a microstimulator and the insertion of electrode wires into the PPF, particularly showing promise in treating chronic cluster headaches (CCH) and refractory trigeminal neuralgia (TN) (5, 6). However, they carry risks such as infection, device failure, or lead displacement. In contrast, non-invasive techniques, which avoid incisions or implants, are generally associated with fewer complications and may be more suitable for long-term management.

Among non-invasive options, acupuncture and electroacupuncture have emerged as promising modalities for SPG intervention, with numerous clinical studies supporting their efficacy. Despite this clinical promise, progressing mechanistic research of acupuncture to the preclinical level faces a major obstacle: the lack of a standardized and reproducible animal model for SPG-targeted acupuncture. The deep anatomical location of the SPG, along with its proximity to critical structures such as the internal carotid artery, foramen rotundum, and pterygoid canal, has not been accurately quantified in commonly used experimental animals (e.g., SD rats). Without such a precise anatomical coordinate system, any targeting of intervention points would be empirical and approximate. Currently, no studies have systematically designed and validated an acupuncture pathway that can precisely and reliably reach the SPG in rats by combining modern imaging and microanatomical techniques. Key parameters such as insertion point, angle, and depth lack evidence-based standardized protocols, which decreases the reliability and reproducibility of experimental results. This greatly limits our ability to investigate the mechanistic effects of SPG acupuncture using animal models. It is crucial to emphasize that inherent craniofacial anatomical differences exist between SD rats and humans. Therefore, the anatomical coordinates and acupuncture parameters established in this rat model are primarily intended to address methodological bottlenecks in preclinical research and cannot be directly extrapolated to human clinical practice. While prior work from our team and others has affirmed the therapeutic value of PPF interventions, the deep mechanisms of acupuncture acting on PPF tissues remain poorly elucidated. Thus, the primary objective of this study is not to propose direct clinical solutions, but to delineate the spatial coordinates of the PPF in SD rats through microanatomical techniques, establish a precise localization system based on bony landmarks, and accordingly design a feasible acupuncture pathway. The accuracy of needle placement will be ultimately verified using real-time imaging.

## 2 Materials and methods

### 2.1 Experimental animals and grouping

This study used 12 SPF-grade adult Sprague–Dawley (SD) rats (6 males and 6 females), aged 6–8 weeks, weighing 200–250 g, provided by SBF (Beijing) Biotechnology Co., Ltd. [Animal Production License No. SCXK (Jing) 2024-0001]. All animals were housed at the Animal Center of Xiyuan Hospital, China Academy of Chinese Medical Sciences, under controlled environmental conditions (temperature: 22 ± 2 °C, humidity: 50 ± 5%), with a 12-h light/dark cycle and free access to standard rodent chow and drinking water. The rats were randomly assigned to 4 groups (*n* = 3/group) using a random number table: the male dissection group, the female dissection group, the male acupuncture group, and the female acupuncture group, with 3 rats in each group. The sample size was guided by the 3R principles (Replacement, Reduction, Refinement) of animal research ethics.

This study was approved by the Ethics Committee of Xiyuan Hospital, China Academy of Chinese Medical Sciences (Approval No. 2024XLCY002-3). All procedures strictly followed the guidelines set by the Ministry of Science and Technology of the People’s Republic of China in the 2006 “Guidelines for the Care and Use of Laboratory Animals,” aiming to minimize animal suffering and stress.

### 2.2 Main reagents and instruments

#### 2.2.1 Main reagents

Anhydrous Ethanol (100092683, China National Pharmaceutical Group Chemical Reagents Co., Ltd.), Xylene (10023418, China National Pharmaceutical Group Chemical Reagents Co., Ltd.), n-Butanol (100052190, China National Pharmaceutical Group Chemical Reagents Co., Ltd.), Environmentally Friendly Dewaxing Solution (G1128, Servicebio), Universal Tissue Fixative Solution (G1101, Servicebio), Hematoxylin and Eosin (H&E) High-Definition Staining Kit (G1076, Servicebio), Neutral Resin (10004160, China National Pharmaceutical Group Chemical Reagents Co., Ltd.).

#### 2.2.2 Main instruments

Stereomicroscope (M80, Leica, Germany), Digital X-ray Fluoroscopy System (Mobilett Mira Max, Siemens, Germany), Digital Caliper (500-196-30, Mitutoyo, Japan), Disposable Sterile Acupuncture Needles (0.25 × 25 mm, Suzhou Huatuo Medical Instruments Co., Ltd.), Ophthalmic Microsurgical Instrument Set (78001, RWD Life Science, Shenzhen, China), Tissue Processor (Donatello, DIAPATH, Italy), Embedding Machine (JB-P5, Junjie Electronics, Wuhan, China), Pathological Sectioning Machine (RM2016, Leica Instruments, Shanghai, China), Cryostat (JB-L5, Junjie Electronics, Wuhan, China), Tissue Spreader (KD-P, Kedi Instruments, Jinhua, Zhejiang, China), Oven (GFL-230, Laibo Rui Instruments, Tianjin, China), Cryostat Sectioning Machine (CRYOSTAR NX50, Thermo Fisher Scientific, China), Adhesive Glass Slides (Paraffin Sections) (G6012-1, Servicebio), Adhesive Glass Slides (Frozen Sections) (G6012-2, Servicebio), Cover Slips (10212432C, Jiangsu Shitai Laboratory Equipment Co., Ltd.), Upright Optical Microscope (Nikon Eclipse E100, Nikon, Japan), Imaging System (Nikon DS-U3, Nikon, Japan).

#### 2.2.3 Data analysis software

Scanner (Pannoramic MIDI, 3DHISTECH, Hungary), Image Acquisition Software (CaseViewer 2.4, Seville Biotechnology Co., Ltd., Wuhan, China).

### 2.3 Anatomical pathway

**Figure 1:**
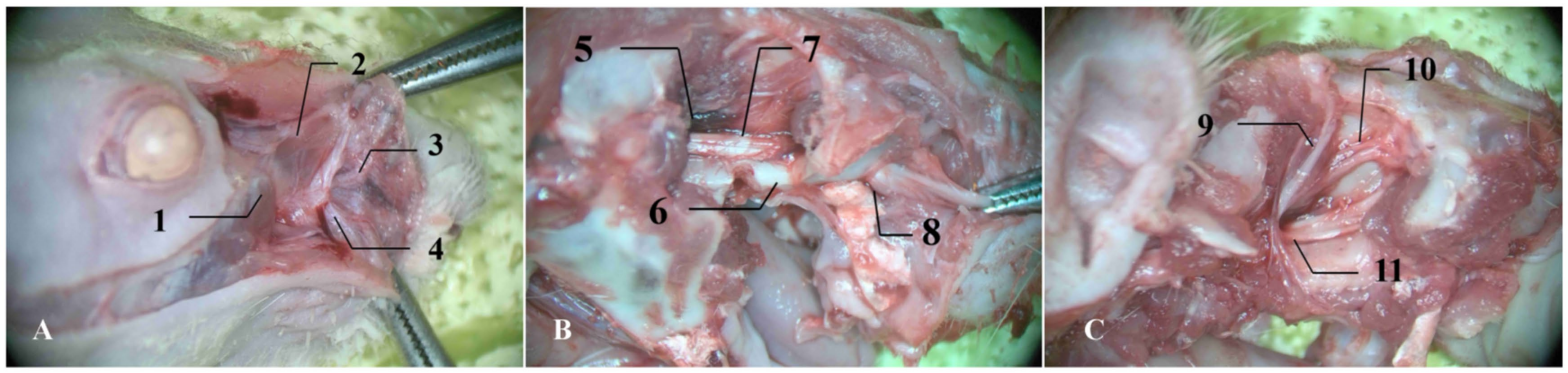
Anatomical landmarks of the right pterygopalatine fossa in SD rats. **(A)** Following blunt dissection of the paranasal subcutaneous tissue, the levator nasolabialis muscle and the external nasal and superior labial branches of the maxillary nerve are exposed. **(B)** After removal of the superficial tissue overlying the maxilla, the maxillary nerve is exposed and traced to the orbital cavity. **(C)** Removal of the orbital contents reveals the fully exposed frontal nerve, ophthalmic nerve (V1), and maxillary nerve (V2), with the sphenopalatine ganglion situated medial to the maxillary nerve. Numerical labels indicate: (1) levator nasolabialis muscle; (2) nasalis muscle; (3) external nasal branch of maxillary nerve; (4) superior labial branch of maxillary nerve; (5) anterior ethmoidal foramen; (6) zygomatic arch; (7) maxillary nerve (main trunk); (8) infraorbital nerve; (9) frontal nerve; (10) ophthalmic nerve; (11) maxillary nerve.

Based on authoritative rat brain anatomical atlases, including *The Rat Brain in Stereotaxic Coordinates*, the anatomical pathway to the PPF was planned, using the right side as an example (Figure 1). The specific procedures were as follows: A longitudinal incision was made 2 mm lateral to the right nostril, parallel to the nasomaxillary suture, to separate the skin of the head and neck on the lateral side towards the caudal direction. After blunt dissection of the subcutaneous tissue, the levator nasolabialis muscle was exposed. The muscle was longitudinally separated along the direction of the muscle fibers and retracted inward to expose the periosteum of the maxilla. After stripping the periosteum from the surface of the maxilla, the maxillary nerve was used as a landmark. The dissection was continued along the dorsolateral edge of the maxilla towards the caudal direction to locate the superficial branch of the infraorbital nerve, and the dissection was extended posteriorly to its exit point at the infraorbital foramen (approximately 3 mm dorsal to the root of the maxillary molars). Using micro bone forceps, the maxilla surrounding the infraorbital foramen was gradually removed while preserving the integrity of the infraorbital canal, and the bone window was enlarged towards the inferior margin of the orbit. Deep within the orbital apex, several large nerves were observed radiating outward. After enucleation of the eyeball and removal of the orbital fat pad and other orbital contents, the optic nerve (CN II), the ophthalmic branch (V1), and the main trunk of the maxillary nerve (V2) were fully exposed. The posterior one-third of the zygomatic arch was further removed with micro-rongeurs to open the PPF. Meticulous dissection of the adipose and vascular plexus within the fossa revealed the main trunk of the maxillary nerve (V2). The SPG was located medial to the main trunk of V2, closely adherent to its medial border and connected to it by short neural roots. The ganglion appeared as a grayish-white, translucent oval nodule, approximately 0.5 mm in diameter. Its caudal end was continuous with the vidian nerve approaching from posteriorly. The SPG, the medial border of the maxillary nerve, and the vidian nerve collectively formed a distinctive “Y-shaped” or “T-shaped” configuration. The identification of these neural structures and their spatial relationships to bony landmarks were verified with reference to published anatomical studies of the rat cranial base (7–12).

### 2.4 Measurement

**Figure 2:**
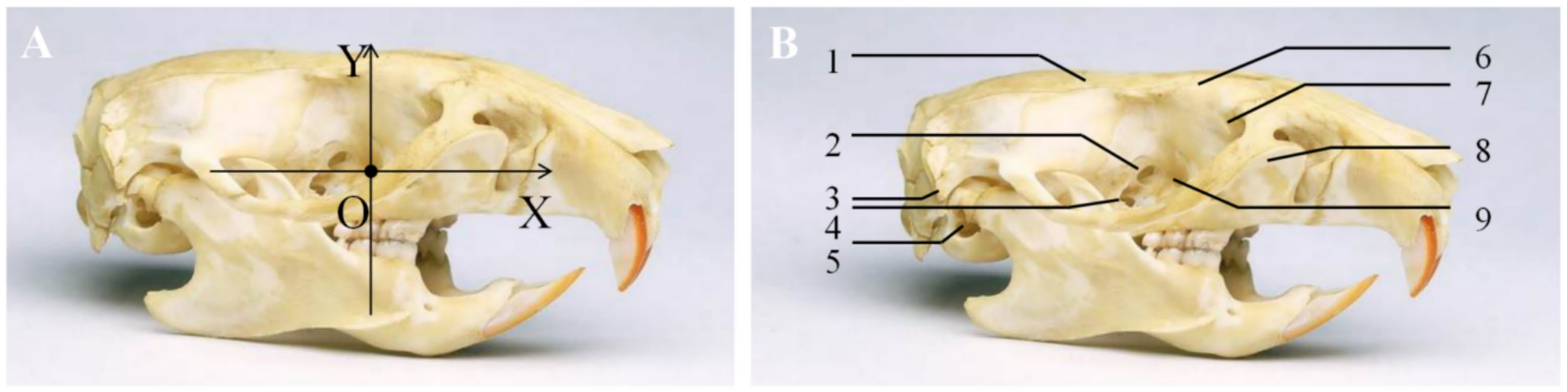
Coordinates of the sphenopalatine ganglion. **(A)** A coordinate system was established with the sphenopalatine foramen as the origin. The *Y*-axis was drawn along the sagittal plane, with the ventral side as negative and the dorsal side as positive. The *X*-axis, perpendicular to the *Y*-axis (in the transverse direction), was drawn with the caudal side as negative and the cranial side as positive. **(B)** (1) Parietal bone; (2) anterior ethmoidal foramen; (3) styloid process; (4) optic foramen; (5) external auditory meatus; (6) frontal bone; (7) lacrimal bone; (8) maxillary bone; (9) sphenopalatine foramen.

The distance between the sphenopalatine ganglion and key anatomical landmarks, including the sphenopalatine foramen, foramen rotundum, and the opening of the pterygoid canal, was measured under a stereomicroscope. A coordinate system was established with the sphenopalatine foramen as the origin (O point). The *Y*-axis was drawn along the sagittal plane, with the ventral side as negative and the dorsal side as positive. The *X*-axis, perpendicular to the *Y*-axis (in the transverse direction), was drawn with the caudal side as negative and the cranial side as positive (Figure 2). The coordinates of the sphenopalatine ganglion were recorded. All anatomical measurements were performed by a researcher who was blinded to the group allocation to reduce potential bias in the data collection process.

### 2.5 Tissue collection

Under deep anesthesia (intraperitoneal injection of 2% pentobarbital sodium, 200 mg/kg) and stereotaxic fixation, the target area was fully exposed via the aforementioned pterygopalatine fossa anatomical approach. Under a stereomicroscope (M80, Leica), using the maxillary nerve (V2) traversing this region and its branches as key anatomical landmarks, typical surrounding structures were identified and confirmed. These included the maxillary nerve, vidian nerve, and the grayish-white translucent nodule located medially (SPG)—thereby delineating the overall boundaries of the target area. Using Vannas-style microscissors, a three-dimensional tissue block was meticulously excised along the natural boundaries of the pterygopalatine fossa. This block contained the maxillary nerve, SPG, residual vidian nerve, sphenopalatine artery, and surrounding loose connective tissue. The completely harvested pterygopalatine fossa tissue block was immediately immersed in 4% paraformaldehyde (4 °C) for 24 h of fixation. After rinsing with PBS, the tissue was dehydrated through a graded ethanol series (75% to absolute ethanol), cleared in xylene, embedded in paraffin, and coronally sectioned into 5 μm continuous slices for subsequent HE staining, Nissl staining, and immunofluorescence staining.

### 2.6 HE/Nissl staining of pterygopalatine fossa tissue

HE staining was performed in sequential steps: hematoxylin–eosin staining, gradient ethanol dehydration, and mounting with neutral gum. Nissl staining was carried out by incubating the tissue with 0.1% thionin blue (pH 4.0) for 10 min, followed by ethanol differentiation, xylene clearing, and mounting. The staining results were used to analyze the morphology of SPG neurons (HE) and the distribution of Nissl bodies (thionin blue). All procedures were performed using a CRYOSTAR NX50 microtome and observations were recorded using a Nikon Eclipse E100 microscope.

### 2.7 Immunofluorescence staining

To enable definitive cellular identification of the acquired pterygopalatine fossa tissue, including its cell types, neural architecture, and status, as well as to assess potential microscopic damage following acupuncture intervention, immunofluorescence staining was performed on its consecutive paraffin-embedded sections. After deparaffinization, rehydration, and heat-induced antigen retrieval in EDTA buffer, the sections were blocked with 5% normal goat serum at room temperature for 30 min to minimize nonspecific binding. Subsequently, the sections were incubated overnight at 4 °C with specific primary antibody cocktails designed to address distinct histological questions: To delineate the overall neural network and Schwann cell structures, a mixture of mouse anti-β-III-tubulin (1:500) and rabbit anti-S100β (1:400) was used to visualize the distribution of neurons and surrounding supportive cells; To investigate potential astrocytic responses, a mixture of mouse anti-β-III-tubulin (1:500) and rabbit anti-GFAP (1:500) was applied, aiming to detect the presence and activation status of any central glial components in this peripheral region. Following thorough washing, the sections were incubated for one hour at room temperature with a corresponding secondary antibody cocktail containing Alexa Fluor 488-conjugated goat anti-mouse IgG (1:400) and Alexa Fluor 594-conjugated goat anti-rabbit IgG (1:400). All nuclei were counterstained with DAPI (1:1,000), and the sections were finally mounted with an anti-fade mounting medium. All stained sections were observed under a confocal microscope, and high-resolution images were acquired for subsequent analysis.

### 2.8 Acupuncture pathway verification

Based on preliminary anatomical results, the acupuncture entry point and pathway for the pterygopalatine fossa region in rats were identified. A digital X-ray fluoroscopy system was used to simultaneously capture lateral and coronal images during acupuncture. The accuracy of the needle tip’s positioning within the pterygopalatine fossa region was verified, and the coordinates of the entry point on the body surface, along with the needle insertion depth, direction, and the angle relative to the coronal plane, were recorded when the needle tip reached the pterygopalatine fossa region.

The acupuncture procedures were performed by a licensed acupuncturist with 10 years of experience in performing acupuncture on animal models. This expertise ensured consistent application of the intervention.

## 3 Results

### 3.1 Comparison of sphenopalatine ganglion localization between the two rat groups

The anatomical regions of the male and female rat groups in the pterygopalatine fossa are located on the lateral side of the vertical plate of the palatine bone, on the medial side of the posterior maxilla (adjacent to the area behind the last molar), in the deep region posterior to the orbit, and within a depression just anterior to the pterygoid process of the sphenoid bone. This area is small and deep in morphology in rats, with closely arranged surrounding structures. The main contents within the fossa include adipose tissue, vascular plexus, and nerve fibers. The spatial positioning of the sphenopalatine ganglion in six SD rats was measured under a stereomicroscope, and the results are presented in Table 1. To assess gender differences, an independent samples *t*-test was performed. The results showed no significant differences in the spatial coordinates of the sphenopalatine ganglion (*X*-axis: males +0.05 ± 0.03 mm, females +0.04 ± 0.01 mm; *Y*-axis: males −0.62 ± 0.08 mm, females −0.54 ± 0.06 mm) between male and female rats (*X*-axis: *p* = 0.613; *Y*-axis: *p* = 0.238). Therefore, gender did not significantly affect the anatomical coordinates.

**Table 1:** Spatial coordinates of the key anatomical landmarks in male/female rat dissection groups.

Structure	Corresponding label in Figure 2B	Group	*X*-axis (mm)	*Y*-axis (mm)	Anatomical location description
Sphenopalatine ganglion (34, 35)	Not shown in Figure 2B	Male rat dissection group	+0.05 ± 0.03	−0.62 ± 0.08	Located just ventrally and slightly laterally to the sphenopalatine foramen, beneath the maxillary nerve (V2)
		Female rat dissection group	+0.04 ± 0.01	−0.54 ± 0.06	
Region analogous to foramen rotundum (V2 exit)	Not explicitly labeled; region dorsal/posterior to SPF	Male rat dissection group	−0.80 ± 0.10	+0.72 ± 0.10	Located dorsolaterally above the sphenopalatine foramen and posteriorly below the optic nerve canal, serving as the entry point for the ophthalmic and maxillary branches of cranial nerves III, IV, VI, and V
		Female rat dissection group	−0.72 ± 0.08	+0.66 ± 0.09	
Optic foramen (36)	4	Male rat dissection group	+3.40 ± 0.20	+1.55 ± 0.15	The largest foramen in the orbit, passing through the orbitosphenoidal part of the anterior sphenoid bone, serving as the pathway for the optic nerve
		Female rat dissection group	+3.28 ± 0.12	+1.46 ± 0.13	
Anterior ethmoidal foramen	2	Male rat dissection group	+3.85 ± 0.22	+1.85 ± 0.18	Located ventrally and anteriorly to the optic nerve canal, serving as the passageway for the ophthalmic branch of the trigeminal nerve
		Female rat dissection group	+3.81 ± 0.18	+1.77 ± 0.17	
Pterygoid canal opening	Not shown in Figure 2B	Male rat dissection group	−1.05 ± 0.15	−1.08 ± 0.12	Located medially on the posterior wall of the pterygopalatine fossa, at the base of the pterygoid process, through which the pterygoid canal nerve exits
		Female rat dissection group	−0.99 ± 0.13	−1.01 ± 0.09	

### 3.2 HE/Nissl staining of pterygopalatine fossa region tissue

**Figure 3:**
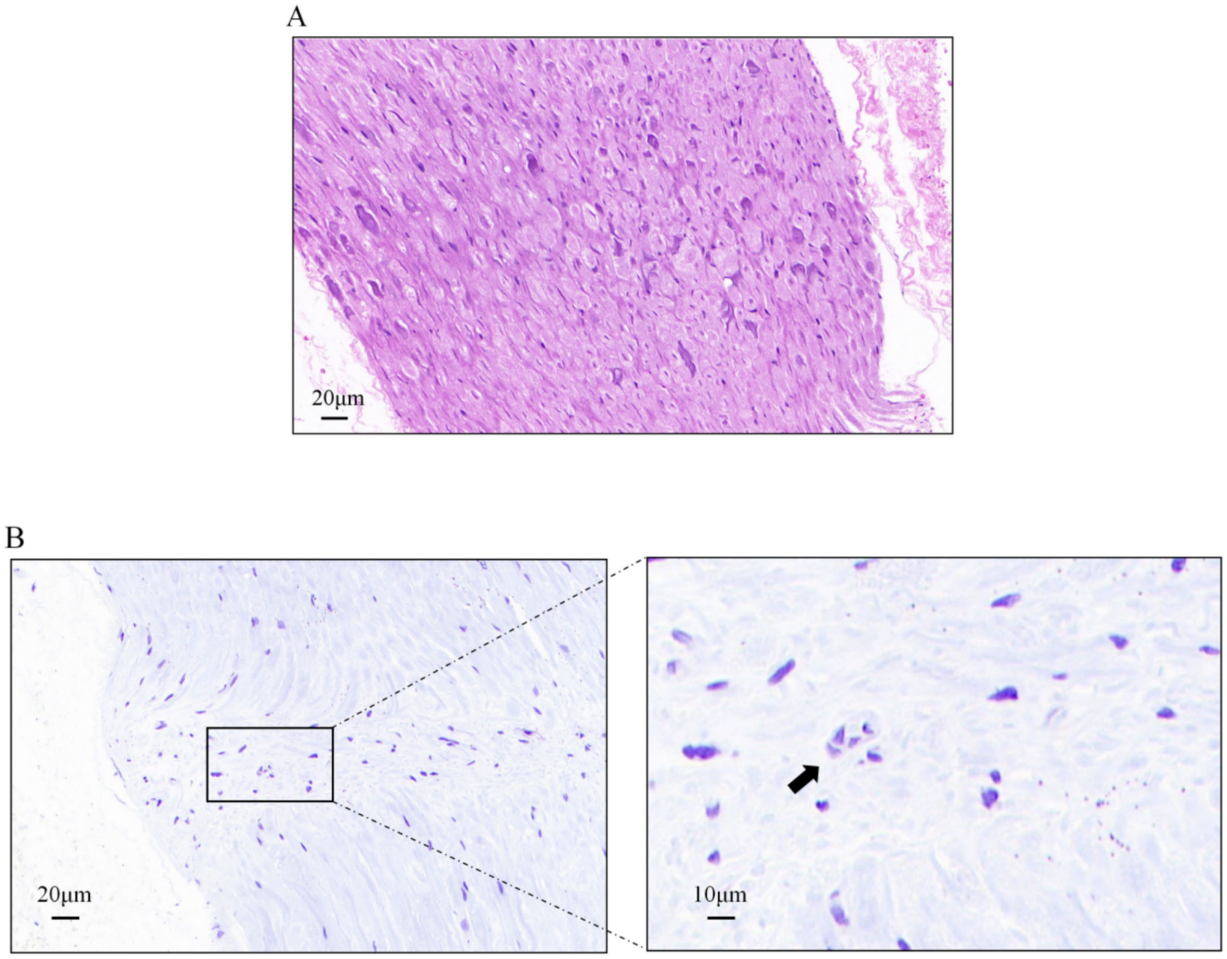
Tissue characteristics of the rat pterygopalatine fossa region. **(A)** HE staining of the neural tissue in the pterygopalatine fossa region (×400), scale bar: 20 μm. **(B)** Nissl staining of the neural tissue in the pterygopalatine fossa region, left image (×400), scale bar: 20 μm; right image (×800), scale bar: 10 μm. The Nissl bodies are indicated by the black arrows.

The results of HE/Nissl staining of the rat pterygopalatine fossa region are shown in Figure 3. HE staining reveals tightly arranged and well-organized tissue cells with intact structure, with neuronal cells aggregating. Nissl staining shows the presence of Nissl bodies.

### 3.3 Immunohistochemical validation of the SPG

**Figure 4:**
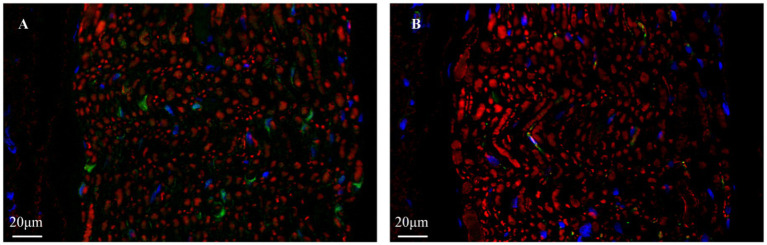
Immunofluorescence characterization of neural tissues in the rat pterygopalatine fossa region. **(A)** Representative image of double immunofluorescence staining for β-III-tubulin (red, neuronal marker) and S100β (green, Schwann cell marker). The red signals reveal a dense network of neuronal fibers and somata. The green signals are scattered and show close spatial association with the neural fiber structures. **(B)** Representative image of double immunofluorescence staining for β-III-tubulin (red) and GFAP (green, astrocyte marker). The β-III-tubulin signal outlines neuronal structures, whereas the GFAP signal was minimal, indicating the absence of notable glial activation or neural stress response in the target region.

To definitively identify the target structures and evaluate the safety of acupuncture intervention at the cellular level, we conducted comprehensive immunofluorescence analysis, with the results presented in Figure 4. Co-staining with the pan-neuronal marker β-III-tubulin and the satellite glial cell marker S100β revealed, as shown in Figure 4A, abundant β-III-tubulin-positive red signals within the acquired tissue field, presenting as distinct circular or oval structures. S100β-positive green signals formed clear ring-like structures enveloping individual or small clusters of red neuronal somata. The co-localization pattern of red nerve fibers and scattered green signals strongly demonstrates that the structural units of nerves within the pterygopalatine fossa remained intact following acupuncture intervention. This organized architecture provides the essential microenvironment for nerve fibers to maintain normal physiological functions, such as nerve impulse conduction. Furthermore, assessment of glial cell activation status via co-localization staining of GFAP and β-III-tubulin showed dense and evenly distributed β-III-tubulin-positive red signals throughout the field. In contrast, GFAP-positive green signals were of very low intensity, barely detectable, with no observed star-shaped, process-rich morphology typical of reactive astrocytes. The immunofluorescence results not only provide conclusive molecular validation of the neural organization in the pterygopalatine fossa region but also confirm that the established acupuncture pathway does not elicit significant glial cell activation, thereby affirming its safety at the microscopic level.

### 3.4 Acupuncture pathway and X-ray verification in the rat pterygopalatine fossa region

**Figure 5:**
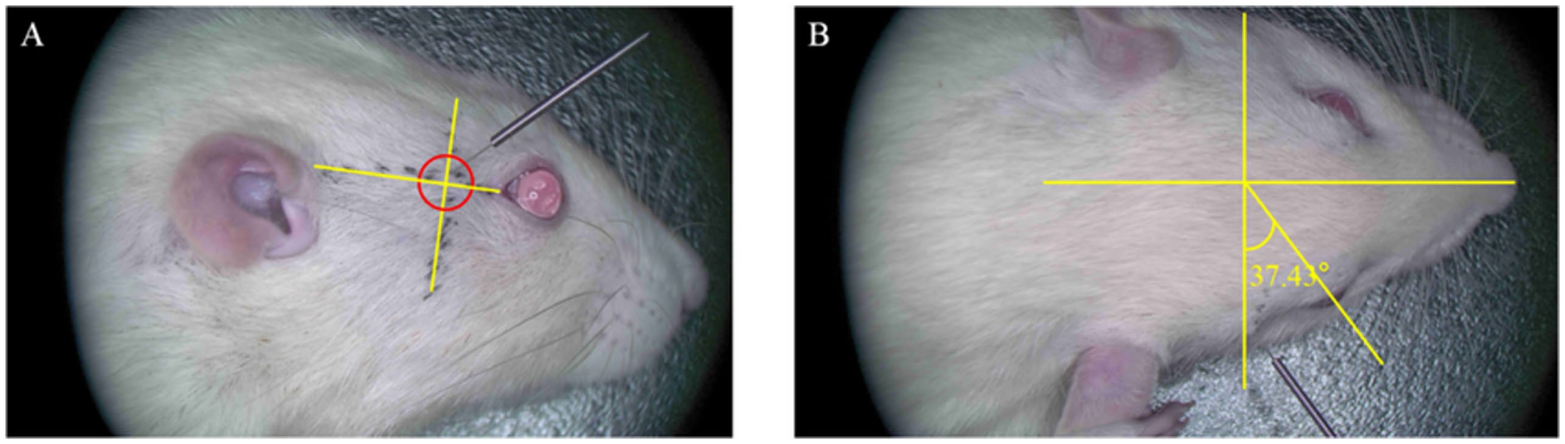
Diagram of needle insertion in the right pterygopalatine fossa region of the rat. **(A)** Lateral view of the rat’s right side. The entry point is located 0.8–1.0 cm from the external canthus along the line connecting the external canthus and the upper edge of the external ear. The needle insertion direction is posteriorly, downward, and medially, with an insertion angle of approximately 45° relative to the horizontal auxiliary line. **(B)** Lateral view of the rat’s head. The needle insertion direction is medially and posteriorly, with an angle of 30–45° relative to the coronal plane of the rat.

Based on preliminary microanatomical and spatial coordinate measurements, we designed and validated an acupuncture pathway for precise targeting of the PPF in SD rats. The needle insertion point was determined along an auxiliary line connecting the lateral canthus of the eye to the superior border of the external auditory meatus. The point was located caudal to the lateral canthus, immediately posterior to the orbital wall (approximately 0.8–1.0 cm from the lateral canthus). The needle was oriented caudally at an angle of approximately 30–45° relative to the sagittal plane and approximately 45° downwards relative to the horizontal plane of the auxiliary line. The direction of insertion was posterior, inferior, and medial. The insertion depth was approximately 1 cm, with the endpoint of the procedure defined as the moment the needle tip encountered the bony resistance of the anterior wall of the PPF (Figure 5). Needle sensation: Upon penetration of the subcutaneous tissue, the practitioner experiences a sensation of “void” as the needle enters. As the needle is advanced towards the target area, a sensation of obstruction is felt when it contacts the periosteum. A slight rotation of the needle produces a “drag” sensation, indicating the needle is in the proper position.

**Figure 6:**
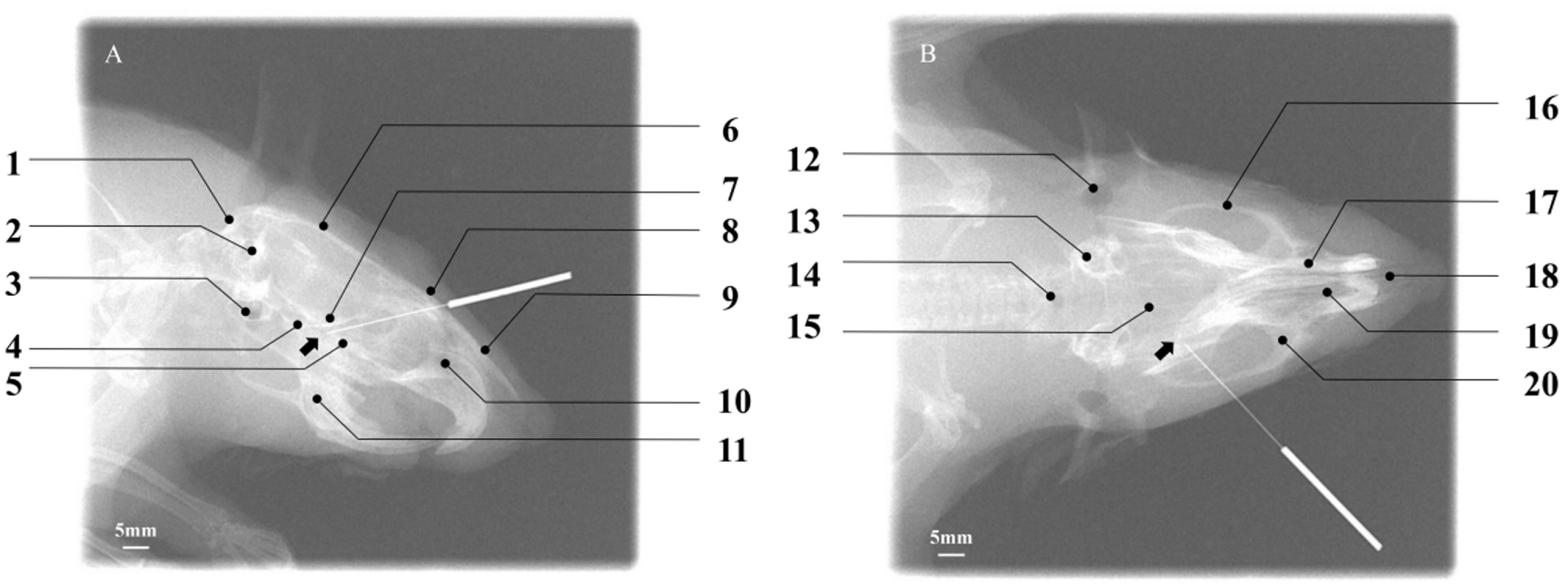
Verification of acupuncture needle placement within the pterygopalatine fossa using digital X-ray fluoroscopy. **(A)** Lateral view: the needle tip (NT) is positioned within the target PPF region, with the black arrow indicating the location of the sphenopalatine foramen. Key cranial landmarks are annotated: (1) occipital bone; (2) foramen magnum; (3) auditory structures/otic region; (4) basisphenoid bone; (5) palatine bone; (6) parietal bone; (7) presphenoid bone; (8) frontal bone; (9) nasal bone; (10) nasal cavity; (11) mandible. Scale bar: 5 mm. **(B)** Coronal view: the NT is located within the target PPF, with the black arrow indicating the sphenopalatine foramen. Annotated cranial landmarks include: (12) external auditory meatus; (13) internal ear; (14) foramen magnum; (15) parietal bone; (16) zygomatic bone; (17) nasal bone; (18) nasal septum; (19) nasal cavity; (20) maxilla. Scale bar: 5 mm. The successful placement, as defined by its spatial relationship to surrounding cranial landmarks, confirms the accuracy of the established acupuncture pathway.

To objectively verify whether the needle tip (NT) accurately reached the PPF region, digital X-ray fluoroscopy was performed on six rats in the acupuncture group for image acquisition and spatial analysis. In the lateral radiograph (Figure 6A), the needle tip was ultimately located within a triangular radiolucent area bounded ventrally by the basisphenoid bone, posteriorly by the maxilla, and dorsally by the perpendicular plate of the palatine bone. This area corresponds precisely to the core projection zone of the PPF in the lateral view. In the coronal radiograph (Figure 6B), the needle tip was clearly observed within a narrow space located lateral to the nasal cavity, medial to the body of the maxilla, and superior to the horizontal plate of the palatine bone. This space is bounded dorsally by the region extending from the inferior orbital fissure and ventrally by the hard palate (formed by the palatine process of the maxilla and the horizontal plate of the palatine bone), accurately corresponding to the anatomical position of the PPF in the coronal plane. The radiographic results confirmed that all needle tips successfully and consistently reached the target area in the PPF, thereby validating the accuracy and reproducibility of the present acupuncture pathway.

## 4 Discussion

### 4.1 Main findings and direct contributions of this study

This study integrated microanatomy, *in vivo* imaging, and histomolecular techniques to systematically establish a precise anatomical coordinate system for the PPF in SD rats for the first time, and successfully designed and validated a standardized acupuncture pathway targeting this region. Through meticulous microdissection, we established a precise spatial coordinate system centered on bony landmarks for the SPG in SD rats. Microanatomical measurements revealed that the SPG was consistently located ventrolateral to the sphenopalatine foramen. While minor numerical differences in spatial coordinates were observed between sexes (male: *X* = +0.05 ± 0.03 mm, *Y* = −0.62 ± 0.08 mm; female: *X* = +0.04 ± 0.01 mm, *Y* = −0.54 ± 0.06 mm), these were not statistically significant. This precise spatial localization not only provides a reliable anatomical foundation for targeted interventions on PPF peripheral nerves but also establishes a critical data base for subsequent research into related disease mechanisms.

Building upon these anatomical findings, we designed a standardized acupuncture pathway and objectively verified its accuracy for the first time using digital X-ray fluoroscopy for spatial, visual confirmation. We optimized the intervention parameters, ultimately defining the core parameters as follows: an insertion point 0.8–1.0 cm from the lateral canthus along the line connecting the lateral canthus to the superior border of the external auditory meatus, immediately posterior to the orbital wall; an insertion angle of 30–45° relative to the sagittal plane and 45° downward relative to the coronal plane; a direction of insertion that is posterior, inferior, and medial; and an insertion depth of approximately 1 cm, with the procedural endpoint defined as the needle tip contacting the bony resistance of the anterior PPF wall accompanied by the characteristic “needle-grasping” sensation. Subsequently, we employed digital X-ray fluoroscopy to objectively verify the endpoint accuracy of this standardized pathway *in vivo* for the first time. Image analysis of six rats in the acupuncture group confirmed that all needle tips accurately and reproducibly reached the target area following the specified parameters. In the lateral view, needle tips were located within the triangular radiolucent area bounded ventrally by the basisphenoid, posteriorly by the maxilla, and dorsally by the perpendicular plate of the palatine bone. In the coronal view, tips were clearly positioned within the narrow space lateral to the nasal cavity and medial to the maxilla. These results confirm that this pathway accurately and reproducibly guides the needle tip to the core area of the PPF containing the SPG. The application of this technique significantly enhances the accuracy and reproducibility of acupuncture interventions in animal experiments, providing crucial technical assurance for obtaining reliable and consistent experimental data.

Following successful precise targeting, we conducted multi-dimensional, molecular-level validation of target identity, intervention safety, and neural activity through a series of histological and immunofluorescence analyses. Conventional HE staining clearly revealed numerous bundles of nerve axons in the PPF region post-intervention, confirming successful sectioning through the major neural pathways within the PPF. Nissl staining observed scattered Nissl bodies. These results preliminarily indicate the presence of a complex neural network in the PPF region and suggest the absence of significant structural damage to peripheral neural tissues caused by this acupuncture method. Co-staining using the pan-neuronal marker β-III-tubulin and the Schwann cell marker S100β clearly delineated the intact architecture of nerve fibers and their supportive environment within the PPF. As shown in Figure 4A, the field contained abundant *β*-III-tubulin-positive red signals, displaying clear bundled nerve fiber morphology. Concurrently, S100β-positive green signals were closely associated with these neural structures, exhibiting typical “encapsulating” or “parallel” distribution patterns. This morphology is a definitive indicator of Schwann cells orderly aligning along neuronal axons to form functional nerve fibers. Furthermore, staining for the astrocyte marker GFAP assessed potential neuroinflammation or tissue stress responses induced by the intervention. Results showed very low, nearly undetectable GFAP signal intensity against the background of the dense β-III-tubulin-positive neural network. Upregulation of GFAP is a key molecular event indicating activation of glial cells (including central astrocytes and peripheral Schwann/satellite glial cells) in response to injury or inflammation. The widespread absence of its signal strongly suggests that the precise acupuncture pathway established in this study is a minimally invasive intervention that does not elicit significant glial stress or neuroinflammatory cascades in the target region. This indicates that the acupuncture method developed is not only anatomically accurate but also exhibits good safety and tolerability at the physiological level, further supporting the feasibility of this intervention protocol for future experimental research.

In conclusion, this study successfully established an acupuncture model targeting the PPF in experimental animals. The establishment of this model provides, for the first time, a robust and reliable methodological tool for in-depth investigation of the neurobiological mechanisms by which peripheral neural modulation in the PPF region influences craniofacial disorders (such as cluster headache and allergic rhinitis) in SD rat models. Although the sample size of this study has certain limitations, it offers new perspectives and a powerful technical pathway for future translational medical research and preclinical pharmacodynamic evaluations in related fields.

### 4.2 Anatomical rationale for the SPG as a therapeutic target

In humans, the PPF is a narrow, cone-shaped space deep within the skull base, housing the largest peripheral parasympathetic ganglion—the SPG—and densely populated with key nerves and vessels that influence multisystem functions in the craniofacial region (13). Multiple bony boundaries collectively form the PPF, providing deep protection for the area while simultaneously presenting a challenge for achieving precise stimulation via acupuncture techniques. In addition to being surrounded by bony structures, the PPF’s proximity to several critical anatomical features increases the risks associated with electrical stimulation. These include the internal carotid artery and cavernous sinus posteriorly; the foramen rotundum and inferior orbital fissure superiorly; and the sphenopalatine foramen medially (14).

The SPG, as the core target structure within the PPF, is a highly specialized peripheral neural integration center. It not only primarily mediates parasympathetic pathways but also contains sympathetic and sensory pathways (15). Within the SPG, neural signals from diverse sources (the central nervous system, sensory endings, the sympathetic system) are received, undergo complex processing and integration, and ultimately generate output signals that regulate various craniofacial functions such as vasodilation/constriction, glandular secretion, and sensory transduction (16). Through this extensive information processing by the SPG, target organs respond according to its commands. Abnormal SPG activity can lead to a series of pathological responses, including parasympathetic overactivity, aberrant sensory input, and abnormal vascular reactions. In humans, these manifest as different clinical symptoms: parasympathetic overactivity results in nasal congestion, profuse watery rhinorrhea, and excessive lacrimation; aberrant sensory input causes migraines, trigeminal neuralgia, and nasal itching (17); and abnormal vascular reactions lead to throbbing headaches, facial flushing, tinnitus, and vertigo.

However, significant differences in craniofacial anatomy exist between SD rats and humans. These differences underscore that the core value of this rat model lies in providing a standardized tool for mechanistic exploration, rather than directly defining human treatment parameters. Firstly, regarding bony structures, the human PPF has well-defined boundaries formed by the sphenoid, maxilla, and palatine bones, and features a distinct foramen rotundum serving as an exclusive conduit for the V2 nerve (maxillary nerve). In contrast, the skull base of SD rats is more simplified and delicate. Their V2 nerve often exits the cranium through a common channel continuous with the inferior orbital fissure, shared with other orbital nerves, and lacks an anatomically separate “foramen rotundum” structure entirely homologous to humans. The “V2 exit” referred to in this study is functionally analogous to the human foramen rotundum but is morphologically distinct. Secondly, concerning the SPG’s own morphology and spatial relationships, the human SPG is typically an irregular, flat triangular or spindle-shaped structure suspended within the adipose tissue of the PPF. Conversely, the SD rat SPG tends to be a compact, oval or spherical nodule approximately 0.5 mm in diameter, with tighter connections to the main trunk of the maxillary nerve and the vidian nerve, resulting in a more “lumped” morphology.

Despite these anatomical disparities, the SD rat model holds irreplaceable value for specific aspects of PPF-targeted research. The primary significance of the precise coordinate system and standardized acupuncture pathway established in this study lies in providing a reliable and reproducible platform for in-depth investigation of the common neurobiological mechanisms underlying acupuncture intervention in the PPF peripheral neural tissues under controlled laboratory conditions. These fundamental biological mechanisms are often highly conserved across mammalian species. Therefore, the direct contribution of this model is to address the long-standing technical bottleneck in preclinical mechanistic studies—“imprecise intervention targets and non-reproducible operations”—rather than aiming to provide a surgical blueprint that can be directly scaled and applied to humans.

### 4.3 Potential mechanisms of acupuncture in modulating peripheral neural functions in the PPF

#### 4.3.1 Neural regulation

The core target of acupuncture in the pterygopalatine fossa region is the sphenopalatine ganglion. As the largest peripheral parasympathetic ganglion in the head and face, as well as a critical sensory nerve integration point, the neural regulation mechanism of the SPG is the fundamental basis for the broad therapeutic effects of acupuncture. Current studies have found that stimulation of the pterygopalatine fossa region can directly alter the membrane potential of the SPG or indirectly stimulate the SPG by activating neurons that have an inhibitory effect on the SPG, thereby improving allergic/non-allergic rhinitis and vasomotor rhinitis by suppressing its excessive activity (18). The essence of its action lies in blocking the vicious cycle of “vascular dilation-glandular hypersecretion-neurogenic inflammation” caused by excessive parasympathetic activation of the nasal region. Furthermore, inhibiting the parasympathetic output of the SPG can also regulate vascular tone and glandular secretion, reduce the release of vasodilators such as vasoactive intestinal peptide (VIP) and nitric oxide (NO), and alleviate nasal congestion. In addition, for dry eye disease with lacrimal gland dysfunction, moderate stimulation of the SPG may promote parasympathetic innervation of the lacrimal glands by activating specific neural circuits or modulating subpopulations of neurons within the SPG, thereby increasing basal tear secretion and exerting a therapeutic effect. This bidirectional regulatory effect reflects the “balancing” characteristic of acupuncture. Acupuncture in the pterygopalatine fossa region can also influence migraine (vascular pain) (19), vasospasm after subarachnoid hemorrhage (20), and ischemic stroke (21) by modulating the SPG’s control over the meningeal blood vessels.

#### 4.3.2 Anti-inflammatory and immune regulation

Neurogenic inflammation is a common pathological basis for several diseases, including migraine and rhinitis. The SPG plays a key role in regulating this process, and acupuncture can effectively suppress neuropeptide-driven inflammatory cascades by modulating the function of the SPG. Acupuncture in the pterygopalatine fossa region can downregulate neuropeptide release, reduce neuropeptide receptor expression, and block neuro-immune cell communication. For example, calcitonin gene-related peptide (CGRP), primarily released by trigeminal sensory fibers, is the strongest vasodilatory peptide known and can induce tissue edema and amplify inflammation. Additionally, key mediators such as substance P (SP) and VIP can also dilate blood vessels, stimulate glandular secretion, and induce symptoms such as itching and sneezing. Studies have found that acupuncture of the SPG significantly reduces the release of CGRP, SP, and VIP in peripheral tissues (nasal mucosa, meninges, trigeminal ganglion) as well as in blood and cerebrospinal fluid (18, 22). On the other hand, inhibiting the SPG reduces neuropeptide receptor expression and downregulates the expression of CGRP receptors (23) and SP receptors (24) on target tissues such as endothelial cells and immune cells, diminishing their sensitivity to neuropeptides. After the reduction in the release of these neuropeptides, mast cell degranulation is further inhibited, which is crucial for alleviating rhinitis symptoms such as itching, sneezing, and mucosal edema. Lv et al. (25) found that electroacupuncture in the pterygopalatine region can inhibit NLRP3-mediated pyroptosis and the imbalance of inflammatory factors, thereby exerting anti-inflammatory effects.

#### 4.3.3 Vascular regulation and hemodynamic changes

The nerves in the pterygopalatine fossa region innervate the nasal mucosal vascular plexus, intracranial blood vessels, inner ear vessels, and lacrimal gland vessels. Acupuncture in this region can precisely regulate local or systemic vascular function, improve microcirculation disorders, and achieve a bidirectional beneficial regulation of vascular tone, blood flow perfusion, and vascular permeability, thus treating various head and face diseases such as migraine, rhinitis, sudden deafness, and even cerebrovascular diseases (4, 26). The core mechanism of acupuncture in regulating vascular function and hemodynamics in the pterygopalatine fossa region is the inhibition of pathological vasodilation. In migraine, rhinitis, and vasomotor headaches, excessive parasympathetic activation mediated by the SPG (VIP/NO) (27) and sensory nerves (CGRP) leads to pathological vasodilation, triggering pulsatile headaches, nasal congestion, and tissue edema. In the early stages of certain diseases, such as the prodromal phase of migraine, early sudden deafness, where vasospasm is present, as well as chronic tissue hypoperfusion states such as atrophic rhinitis or reperfusion injury, acupuncture can exert a bidirectional regulatory effect to alleviate pathological vasoconstriction (28).

### 4.4 Study limitations and future perspectives

While this study successfully established a precise anatomical coordinate system and a reproducible acupuncture pathway for the PPF in SD rats, several limitations must be acknowledged. First, although our anatomical delineation primarily relied on bony landmarks and was corroborated at the cellular identity level via serial staining, the incorporation of neural tract-tracing techniques would further strengthen the evidence. The use of tracers such as tetramethylrhodamine-labeled dextran was beyond the scope of this methodological investigation due to the required extended animal survival periods and associated technical risks; however, this remains a crucial direction for future dynamic visualization of SPG connectivity with adjacent nerves. Second, and most notably, this study is primarily methodological in foundation and consequently lacks functional validation of the acupuncture intervention. As data were not acquired in disease models (e.g., cluster headache or allergic rhinitis) and behavioral or physiological readouts (e.g., pain thresholds, nasal symptoms) were absent, the therapeutic efficacy of this precise targeting cannot be inferred from the current dataset. This constitutes the most critical subsequent research direction. Third, the established coordinates and pathway are specific to SD rats. The significant differences in craniofacial morphology between rodents and humans necessitate cautious interpretation regarding the direct clinical translatability of our findings. The immediate value of this work lies in providing a standardized preclinical tool for mechanistic exploration, not in defining human treatment parameters. Finally, while our histological evidence demonstrates structural integrity and an absence of apoptosis or glial activation, more refined quantitative histomorphometric analyses could provide deeper insights into the tissue response.

Despite these limitations, the present model establishes a robust platform for future research. The immediate next step involves applying this precise targeting methodology within established preclinical models of craniofacial pain and rhinitis to quantitatively evaluate its functional outcomes and underlying mechanisms. Furthermore, this platform enables the investigation of key neurobiological questions, such as how PPF acupuncture modulates the release of specific neuropeptides (e.g., CGRP, Substance P, VIP) and influences central nervous system activity. In the long term, insights gained through the application of this standardized method in rigorously controlled animal studies will provide the necessary scientific foundation for optimizing PPF-targeted acupuncture therapies and translating them into more effective, evidence-based clinical applications.

## 5 Conclusion

The sphenopalatine ganglion (SPG) is a critical regulatory target for head and face diseases such as cluster headaches, allergic rhinitis, and cerebrovascular ischemia. Several intervention methods are currently available to modulate the SPG, including SPG radiofrequency ablation (29, 30), implanted stimulators (31, 32), and drug injections (33). Acupuncture/electroacupuncture, as a distinctive technique in traditional Chinese medicine, offers advantages over invasive Western medical procedures due to its non-invasive nature and low cost, and it has shown good clinical efficacy in treating various head and face disorders. However, to further understand the potential mechanisms of acupuncture in treating diseases in the pterygopalatine fossa region, rigorous animal acupuncture models are still required. To date, no studies have clearly defined the detailed pathway of acupuncture in the pterygopalatine fossa, and the SPG in animals lacks standardized localization. This study, through microanatomical localization and imaging verification, for the first time systematically established a spatial anatomical coordinate system for the PPF in SD rats and successfully designed and validated the precise acupuncture pathway to the SPG. At the anatomical level, this study clarified the spatial position of the rat SPG (male: *X* = +0.05 ± 0.03 mm, *Y* = −0.62 ± 0.08 mm; female: *X* = +0.04 ± 0.01 mm, *Y* = −0.54 ± 0.06 mm), and quantified its adjacency to key structures such as the foramen rotundum and pterygoid canal opening, filling the gap in fine anatomical data for the PPF region in small animals. Additionally, a reproducible acupuncture pathway was proposed (entry point: 0.8–1.0 cm caudal to the external canthus; angle: 30–45° in the sagittal plane, 45° in the coronal plane; depth: 1 cm), and real-time verification was achieved using digital X-ray fluoroscopy, providing a standardized protocol for precise intervention in animal experiments.

## Data Availability

The original contributions presented in the study are included in the article/supplementary material, further inquiries can be directed to the corresponding author.

## Glossary

SPG: Sphenopalatine ganglion
PPF: Pterygopalatine fossa
CCH: Chronic cluster headaches
TN: Trigeminal neuralgia
CN II: Optic nerve
V1: Ophthalmic branch of the trigeminal nerve
V2: Maxillary nerve
HE: Hematoxylin–eosin
NT: Needle tip
VIP: Vasoactive intestinal peptide
NO: Nitric oxide
CGRP: Calcitonin gene-related peptide
SP: Substance P
SPF: Specific pathogen free
SD: Sprague–Dawley
PBS: Phosphate-buffered saline
EDTA: Ethylenediaminetetraacetic acid
DAPI: 4′,6-Diamidino-2-phenylindole
IgG: Immunoglobulin G
NLRP3: NOD-like receptor pyrin domain-containing protein 3
MR: Magnetic resonance
